# Falsetto Voice in the Male

**Published:** 1899-04

**Authors:** G. Hudson Makuen

**Affiliations:** Philadelphia, Professor of Defects of Speech in Philadelphia Polyclinic and College for Graduates in Medicine; Laryngologist to St. Mary’s Hospital and the Frederick Douglas Memorial Hospital


### Falsetto Voice in the Male.

Report of five cases—Presented to the Section on Laryngology and Otology at the Forty ninth Annual Meeting of the American Medical Association, held at Denver, Col., June 7-10, 1898.

BY G. HUDSON MAKUEN, M.D., PHILADELPHIA.

Professor of Defects of Speech in Philadelphia Polyclinic and College for Graduates in Medicine; Laryngologist to St. Mary's Hospital and the Frederick Douglas Memorial Hospital.

The falsetto voice, as its name indicates, is essentially a false voice. Its chief characteristics are a high pitch and a thin, piping quality. Its resonance is limited to the region of the head, and therefore it lacks volume and strength. There is also a manifest effort in its production which makes it appear forced and unnatural. The labor seems altogether out of proportion to the results attained. It suggests weakness and a lack of muscular control. It is somewhat characteristic of the eunuch, and therefore it has been called the eunuchoid voice. This quality is not uncommon in the female, whose larynx is comparatively small and delicate and whose nervous organization is often highly tensioned, but it is rare, and therefore more noticeable, in the male. In an experience of more than twenty years as a vocal teacher, not more than ten or twelve cases have come under my observation, and only five of these have reported to me for examination and treatment. A rather close study of these cases, both externally to the larynx and internally by means of the laryngoscope, has revealed some interesting facts as to the nature and cause of this peculiar affliction, for so I think it must be regarded. In the first place, viewed from without, we find the larnyx in an abnormally high position during the production of this voice. The high position is brought about by a faulty action or a faulty co-ordination of the extrinsic muscles of the larynx. These extrinsic muscles may be divided into two sets and designated respectively the levator laryngei and the depressor laryngei.

In the production of the normal voice there is a contention constantly going on between these two sets of muscles, and thus the larynx is held nicely poised in the correct position, and more or less firmly fixed against the spine for each tone of voice and for each syllable of speech. If this natural poise of the larnyx in the correct position be interfered with in any way, there will be an immediate change both in the character or timbre and in the pitch of the voice. The change in character is due to the change in certain resonators of the voice, and the change in pitch to the varying conditions of the vocal ligaments. If, for instance, the larynx be pulled down below its normal position either by the over-action of the depressor or the under-action of the levator muscles, the voice will be heavy and sepulchral in character and unnaturally low in pitch; and if, on the other hand, the larnyx be pulled high up in the throat by a reverse action of the extrinsic muscles, we shall have a thin, high-pitched voice, and if at the same time there be a looseness of the intrinsic muscles the resonance will be impaired and the result will be the falsetto or eunuchoid voice.

It will be observed that this quality of voice is due not only to the high position of the larnyx, but also to the consequent unnatural adjustment of the cartilages of the larnyx and to the effect which this adjustment has upon the vocal ligaments. Another important factor is probably a lack of rigidity or contractility in the intrinsic muscles of the larnyx, thus cutting off the resonance which comes from these muscles and the surrounding parts to which they are attached.

My first case was a young clergyman, who affected this quality of voice because it seemed to him more soft and agreeable than the resonant chest tone. So strongly and favorably did it appeal to his own ear and to his sense of the fitness of things that he was not persuaded to give it up, although it would have been quite possible for him to do so with a little effort and training. In fact, he could produce the true chest voice, but he said it seemed hoarse to him and he preferred not to make the change; and thus was illustrated the old adage: "You may lead a horse to water but you can't make him drink."

My second case was referred to me by a well-known laryngologist in Philadelphia. The patient was 15 years of age, and a tinsmith by trade. At about the time of puberty he went in bathing while overheated and developed a severe laryngitis and lost his voice almost entirely. The usual remedies were applied, and the only sound he was able to produce afterward was that of the falsetto character. Local applications were made to the larynx for a long time, and electricity was applied in all its various forms, but with no effect whatever upon the character of the voice. It took but a short time to discover that he did not appreciate the fact that his voice was different from that of his fellows. He had been told that it was peculiar, but it sounded all right to him. After two or three visits, at each of which he was given various exercises to correct his errors in voice production, he was enabled to sound a good chest note, and when he succeeded in getting it, he said, in a clear resonant voice: "Is that the way you want me to talk?" I said, "Yes;" and he said, "Well, I think I can talk in that way now all the time," and I suppose he did, for he never appeared at my office again.

My next case was a young man 17 years of age, referred to me by Dr. Max Thorner, of Cincinnati. The patient had always been strong and vigorous, except on two occasions, when he had pneumonia—the first attack at 13 and the second at 14 years of age. When he was convalescing from the latter attack the larnyx underwent the change which is usual at that period of life, and the voice presented a peculiar and unusual character. Instead of being a combination of the chest and falsetto tones, the former breaking into the latter, as is common in the male voice at puberty, the chest tone was absent entirely, and in its place there was complete aphonia. The voice was a mixture of a whisper with the falsetto tone, the one breaking into the other in a most curious manner and at the most unexpected times.

The patient usually began speaking in a whisper, which by very great effort he soon forced up into a peculiar falsetto squeak, which seemed ludicrous to those not appreciating the serious aspect of the case. At times it was with great difficulty that he could even whisper or make himself understood at all, and, being an exceedingly bright and intelligent youth, his affliction bore the more heavily upon his mind and made the past three years of his life very miserable. The young man comes from the South, where the humorous side of life is well developed, and his friends at first regarded the whole matter as a huge joke, called him a gosling, and advised him to go back to long dresses. His nervous system was somewhat impaired by his recent illness, and his larynx underwent a greater change than is usual—for it is now a very large one—and he was unable to adjust the various parts of the vocal machinery to the new and varying conditions. Three things, therefore, may have combined to bring about this unfortunate state of affairs: an impaired physical condition, a sudden and unusual increase in the size of the larynx, and a nervous anxiety brought on by the sportive jeers of his friends. Soon after the onset of the trouble he had various minor operations performed in the throat and nose. Hypertrophied faucial tonsils were reduced and a spur was removed from the nasal septum, but no improvement in voice followed.

He was seen first by me in September of last year. A regular course of breathing exercises was instituted for the development of the respiratory muscles, and all attempts at speech were for a time interdicted. There was a marked tremor of all the organs of speech. The pharynx was cocainized and a careful laryngoscopic examination revealed nothing unusual except that the larynx was somewhat above the average size. The vocal cords overlapped both anteriorly and posteriorly in the falsetto tone, and the glottis presented the usual elliptic shape in the aphonic condition. It was observed that during phonation there was a strong contraction of the palato-pharyngei muscles, drawing the larynx high up in the throat. To counteract this up-pulling of the larynx, the tip of the index finger was placed on the dorsum of the tongue, well back against the anterior surface of the epiglottis and firm downward pressure made, and at the same time with the right index finger in the groove on the upper border of the thyroid cartilage externally the larynx was held in a fixed position and the patient was told to say "ah," which he succeeded in doing in a low chest tone for the first time in his life. After several repetitions of the tone with the larynx held for him in this position he was able to control it himself, and after two weeks' faithful practice he went home with a full, rich chest tone which he used in speech with remarkable facility, while it was with great difficulty that he could imitate his former mode of speech.

The next case was a boy of 15 years of age, referred to me by Dr. John B. Roberts, of Philadelphia. His aunt, who came with him, said he has always talked like a girl, and that sometimes his voice would fail him entirely and he could not talk at all; that he was a very nervous boy and came from a nervous and consumptive family. At 7 years of age he had measles, complicated by a very severe attack of la grippe. He had high temperature and was delirious for some time. He is now weak and nervous, and complains a great deal of headache. He fainted during the throat examination. Like the last case, the larynx was found to be pulled up high in the throat by a kind of spasmodic contraction and hardening of the levator laryngei muscles. The muscles under the chin, the genio-hyoid and hyo-glossus were almost as hard as it is possible to make the biceps by flexing the forearm, while the depressor laryngei were weak and flabby. He had some slight tonsillar hypertrophy, which was reduced, and he was put on a general tonic treatment. In addition to this, he was put on a course of what we call laryngeal muscle training by methods which I shall describe briefly after referring to my fifth case.

This boy was 15 years of age. He was nervous and by no means strong. His maternal grandmother and uncle and his paternal grandfather and uncle had died with consumption. His voice was normal until about the time of puberty, when he took a severe cold. It then seemed rough and harsh to him, and he tried to hold it back, with the result of acquiring the falsetto voice. Like Case No. 2, he himself was quite satisfied with his voice, and he said it sounded better to him than the average voice he heard. His father thought it was his own fault that he did not speak as other people. Upon examination it was found that the adjustment of the cartilages was very unusual. The thyroid cartilage was tilted forward and downward so that the pomum Adami was very prominent, and the thyroid rested upon the upper border of the cricoid cartilage, and the two appeared to be united anteriorly. They could not be separated, and there was no crico-thyroid notch.

In some respects the last two cases were similar. They were of the same age; they were both of the neurasthenic type, and they both inherited a tubercular taint. The onset of the trouble was different, however, the former having always used the falsetto voice, while the latter acquired it at the age of puberty. The treatment was similar for both. In fact, the same general plan of treatment was followed in all these cases, and the results were prompt and entirely satisfactory.

Let me explain the plan of treatment. In the first place the general physical condition of the patient was looked into, and nervine tonics were indicated in every case. Then the nose and throat conditions were looked after, and local treatment was resorted to pretty generally, and slight surgical interference in three of the cases. These things are all important and necessary, but not in themselves sufficient to correct the defect. They are only adjuvant to the thing that cures, viz: muscle training. The direct cause of the difficulty was found to be in every case a faulty co-ordination of certain laryngeal muscles, and this faulty co-ordination was mainly extrinsic to the larynx. The extrinsic muscles were not uniformly developed. The levator laryngei were overdeveloped while the depressors were underdeveloped and there was a lack of proper laryngeal balance. The weak muscle was singled out and trained into right action. The best way to do this is to teach the voluntary control of this musc e, and then, by repeated contractions and relaxations, the patient may strengthen it to do its normal work, and thus acquire a harmonious action of the laryngeal muscles. Massage of the larynx was also helpful in restoring the balance and harmonious action of these muscles.—*Am. Med. Ass'n Journal.*

